# High entropy alloys as a bold step forward in alloy development

**DOI:** 10.1038/s41467-019-09700-1

**Published:** 2019-04-18

**Authors:** D. B. Miracle

**Affiliations:** Air Force Research Laboratory, Materials and Manufacturing Directorate, Wright-Patterson AFB, OH 45433 USA

**Keywords:** Mechanical properties, Metals and alloys

## Abstract

Diluting a base element with small amounts of another has served as the basis for developing alloys for thousands of years since the advent of bronze. Today, a fundamentally new idea where alloys have no single dominant element is giving new traction to materials discovery.

## The historical approach to alloy development

Over 5000 years ago, the first alloy developer diluted elemental copper with a small amount of tin to make bronze. An immediate success, this simple alloying approach changed the course of civilizations—and humanity has used the same approach to develop alloys ever since. The idea is simple: an element with some attractive properties is used as a base, and minor amounts of other elements are added to improve the properties. While fewer than a dozen metallic elements were known before the 18th century, limiting options for early alloy developers, the remaining elements were subsequently discovered in quick succession and the best have now all been exploited.

This lightly-alloyed-base-element method has been widely successful, and has become increasingly sophisticated^1^. Alloy additions are now controlled to tenths or hundredths of a percent and alloys can have as many as a dozen minor elements. But all of these alloys still have a single dominant base element.

Bronze signaled the end of the stone age, and the iron age (along with the ability to control small carbon additions to make steel) marked the end of the bronze age. By controlling carbon and impurity elements in large batches, the mass production of cheap steel in the mid-1800s sparked the industrial revolution. In the 20th century, lightly alloyed base elements provided lightweight and high temperature alloys essential for new aerospace industries, fueling additional societal transformations through air transportation, communication and navigation via satellites and GPS.

However, there are signs that this millennia-old approach may be reaching limits. Decades-long, intensive international efforts to develop new materials are failing. These include efforts towards improved high temperature alloys for energy production and aerospace propulsion and low density, low cost alloys for transportation. A major issue behind the missing breakthroughs is the lack of new base elements—no new stable metallic elements have been discovered in nearly 100 years. As a result, materials development is sometimes labeled as a ‘mature technology’. However, a technology is mature only when it runs out of ideas, and a fundamentally new idea has recently appeared that can reinvigorate the discovery of society-changing materials.

## High entropy alloys—a new idea

High entropy alloys (HEAs) were founded by two major ideas from two different research groups with two different motivations, both published in 2004^2,3^. Both ideas focus on the unexplored central regions of multi-element phase diagrams, where all alloy elements are concentrated and there is no obvious base element. Initially emphasizing single-phase, solid solution metallic alloys, the field has grown to include intermetallic and ceramic compounds, as well as microstructures with any number and any type of these phases^4^. HEAs were originally defined as a blend of 5 or more elements with concentrations between 5 and 35 atom percent^3^, but the field now includes materials with as few as 3 principal elements, and where the maximum element concentration may be higher than 35 atom percent. New terms such as complex, concentrated alloys (CCAs)^4^ and high entropy materials (HEMs) embrace these new concepts.

Each HEA is a new alloy base, since each HEA can be modified by minor elemental additions as for current element-based alloys^5^. HEAs give a vast number of new alloy bases. The number of HEA systems (unique combination of elements, without specifying composition) is:

1 $$C\left( {\begin{array}{*{20}{c}} n \\ r \end{array}} \right) = \frac{{n!}}{{r!\left( {n - r} \right)!}}$$

where *n* is the number of elements in the palette from which *r* principal alloy elements are selected. There are *n* = 75 stable elements that aren’t toxic, radioactive or noble gases, giving over 219 million new CCA systems with 3 ≤ *r* ≤ 6 principal elements. However, each new CCA system has many different bases (a base is a unique combination of principal elements and concentrations). For example, if every 10% change in composition gives a distinct CCA base, then a 5-component system will have 906 different CCA bases^1^. Combined with the number of systems, this gives over 592 billion new CCA bases with 3–6 principal elements.

Beyond a cosmic number of new alloy bases to explore, new physical phenomena and exceptional properties are being reported for HEAs and CCAs. Important findings include highly corrugated partial dislocations^6^ that may alter mechanical properties; magnetic hardening—a new strengthening mechanism^7^; exceptional strength, ductility and fracture toughness at cryogenic temperatures^8^; parabolic oxidation in refractory metal CCAs (RCCAs) with kinetics that are two orders of magnitude lower than conventional refractory alloys^9^; exceptional irradiation resistance with self-healing as a proposed mechanism^10^; and new opportunities to fill blank spots in property space^11^.

Initially focused on metals, HEAs now include intermetallic compounds and technical ceramics with a wide range of compelling functional properties, including the thermoelectric effect, piezoelectricity, photovoltaic conversion and shape memory, among others. Ceramic materials also offer structural properties at very high temperatures. These compounds typically have two or three crystal sublattices, where each sublattice is dominated by a single species. Conventional alloying strategies for these materials are often limited to only one elemental substitution on one or two sublattices at a time, and are usually further restricted to the small number of elements that form the same crystal structure as the host. However, HEA studies show that many elements can be substituted on a single sublattice, and that a host structure can be retained even when adding elements that form different crystal structures^12^. With more principal elements than sublattices, HEA concepts can significantly expand conventional alloying approaches for functional materials^13^.

## New challenges

While the promise is great, exploring the enormous number of HEA and CCA compositions and their microstructures is currently the biggest challenge. Exploration must accelerate by 6–9 orders of magnitude to match the explosion in new alloy bases. The Materials Genome Initiative^14^ is already making progress, but with a goal of halving the time to develop materials, it won’t go nearly far enough. New high throughput experiments are needed, especially for structural materials. And new strategies are needed to quickly navigate the winding, narrow path between properties that depend on composition, and those that depend sensitively on both composition and exquisitely designed microstructures^15–17^.

Acquiring new fundamental data is also essential. Current scientific efforts are built on data and knowledge collected more than 50 years ago. This includes thermodynamic data, phase equilibria, phase transformations and phase stability; defects and defect energies; diffusion data and kinetic models; deformation mechanisms under different loading conditions; and the influence of composition on all of these properties. These data are typically available only for materials with a single dominant solvent and provide limited benefit to CCAs.

To illustrate this point, the ordered B2 intermetallic precipitate may be an important strengthening phase in RCCAs. It’s found in a number of these alloys, and the exploration of alloys containing this phase could be accelerated using the CALculated PHAse Diagram (CALPHAD) method. However, its presence can’t be predicted in RCCAs because the B2 phase is essentially absent in binary phase diagrams that involve refractory metals, and so CALPHAD contains neither data nor thermodynamic models for refractory metal B2 phases. New data documenting observed B2 compositions and formation enthalpies in RCCAs, and thermodynamic models built on this data, are essential for continued progress in this direction.

Today, the ability to allocate resources to collect fundamental data is becoming increasingly difficult and relies on convincing funding agencies, publishers, and the community of the importance of doing so. The current lack of data for new physical models and new predictive capabilities in CCAs is a barrier to future scientific progress and collecting this data is expected to lead to new scientific discoveries. Consider, for example, the highly cited original work in high impact journals measuring the mechanical properties of CoCrFeMnNi and CoCrNi at cryogenic temperatures^8,18^ or the measurement of chemical short-range order in Al_1.3_CoCrCuFeNi^19^. Other surprises may be expected in measuring thermodynamic data or diffusion data or elastic constants or defect structures. By remembering the essential role of fundamental data as a major scientific contribution in its own right, and as a springboard for new scientific discoveries, the materials science community is remembering our past to inspire future progress.

High throughput screening tests are an essential part of this strategy. By quickly illustrating trends over broad search spaces, these tests efficiently focus valuable resources on the most promising alloys. Nevertheless, these tests often bring a higher degree of uncertainty than conventional tests, while more accurate, current approaches are slower and have an inherent risk by being able to evaluate only a miniscule fraction of the possible alloys. Classroom students taking a timed test intuitively know how to think about this conundrum—is it better to spend all of the time answering 1 question with 95% confidence, or answering all of the questions with 70% confidence?

Finally, we must begin to think differently about the scope of CCA studies. A small handful of equiatomic or near-equiatomic compositions are commonly used as a proxy for all the alloys in a multi-component phase diagram. We need to explore a broader range of compositions in promising alloy systems. After nearly 15 years of study, the field has explored only seven new alloy families, each based on a palette of similar atoms^4^. While this is a notable accomplishment, we have barely scratched the surface. These groupings of similar elements represent a form of ‘linear thinking’^20^, and we need to create new alloy systems with uncommon element groupings. Like elemental genetic algorithms, these new alloy families may provide unexpected results leading to the society-jarring transformations sought by the materials exploration and development community.
